# A pilot study combining primary busulfan-based haploidentical stem cell transplantation with GD2 antibody to treat high-risk neuroblastoma

**DOI:** 10.1038/s41409-026-02912-2

**Published:** 2026-05-29

**Authors:** Sveva Castelli, Tim Flaadt, Franziska Schulze, Theresa M. Thole-Kliesch, Felix Zirngibl, Lena Oevermann, Annette Künkele, Kathy Astrahantseff, Stefanie Schulte, Louisa Duell, Joerg Fuchs, Thorsten Simon, Barbara Hero, Patrick Hundsdoerfer, Julian M. M. Rogasch, Peter Lang, Steven Warmann, Arend von Stackelberg, Johannes H. Schulte, Angelika Eggert, Hedwig E. Deubzer

**Affiliations:** 1 Department of Pediatric Oncology and Hematology, Charité – Universitätsmedizin Berlin, Berlin, Germany; 2 Department of Pediatric Oncology and Hematology, University Children’s Hospital Tübingen, Tübingen, Germany; 3 Berlin Institute of Health (BIH) at Charité – Universitätsmedizin Berlin, Berlin, Germany; 4 German Cancer Consortium (DKTK), Partner Sites Berlin and Tübingen and German Cancer Research Center (DKFZ), Center, Germany; 5 National Center for Tumor Diseases (NCT), NCT Berlin, a partnership between DKFZ, Charité – Universitätsmedizin Berlin, Berlin Institute of Health at Charité (BIH), Max Delbrück Center, Center, Germany; 6 Department of Pediatric Surgery and Pediatric Urology, University Children’s Hospital Tuebingen, Tuebingen, Germany; 7 Department of Pediatric Oncology and Hematology, University of Cologne, Cologne, Germany; 8 Helios Klinikum Berlin Buch, Berlin, Germany; 9 Department of Nuclear Medicine, Charité – Universitätsmedizin Berlin, Berlin, Germany; 10 Department of Pediatric Surgery and Pediatric Urology, Charité – Universitätsmedizin Berlin, Berlin, Germany; 11 University Hospital Essen, Essen, Germany

**Keywords:** Paediatrics, Stem-cell research

## Abstract

Very high-risk neuroblastoma is induction-refractory and often harbors mutations in RAS and/or p53 signaling combined with telomere maintenance mechanisms. Event-free survival is <20% in these children. Patients unable to mobilize sufficient hematopoietic stem cells to harvest for busulfan/melphalan-based high-dose chemotherapy before autologous transplantation are also at high risk for relapse. Haploidentical stem cell transplantation (haplo-SCT), offering graft-versus-tumor effects and enhanced antibody-dependent cellular cytotoxicity, has emerged as a feasible treatment. We report administration of a conditioning regimen combining myeloablative busulfan/melphalan anti-tumor therapy with the immunological advantages of haplo-SCT and GD2-directed antibody-based immunotherapy with dinutuximab beta (DB). A 5-patient pilot cohort was treated with systemic induction (salvage) therapy and local therapy per national guidelines. Prior to busulfan, melphalan, fludarabine and antithymocyte globulin conditioning followed by T/B-cell-depleted haplo-SCT and 6 DB cycles, 2 patients received [^131^I]MIBG therapy. All patients were successfully engrafted. Three of five patients are alive and have remained in first complete remission for 7.3, 6.3 and 1.5 years after haplo-SCT, while two patients experienced events (one relapse, one non-relapse death). Primary busulfan-based haplo-SCT combined with DB immunotherapy was feasible and effective. Early results suggest a survival benefit for these pediatric patient subgroups at very high risk. Confirmation in a larger controlled trial is warranted.

## Introduction

Neuroblastoma, the most common extracranial solid tumor in pediatric patients, causes 12% of childhood cancer deaths. International Neuroblastoma Risk Group Criteria [1, 2] clearly define three risk groups. Event-free survival reaches only ~50% in patients with high-risk neuroblastoma despite intensive multimodal therapy comprising chemotherapy, surgery, salvage chemoimmunotherapy, high-dose chemotherapy followed by autologous stem cell rescue, radiotherapy and maintenance therapy with GD2-directed immunotherapy combined with retinoic acid [3]. Most relapses of high-risk disease occur within 2 years of diagnosis [4], yielding a 20% overall survival at 4 years [5] and a median progression-free survival of 6.4 months [6].

Important insights into high-risk disease biology have been provided by next-generation sequencing of primary neuroblastomas in the past 10 years [7–10]. Patients with tumors harboring mutations in RAS and/or TP53 signaling combined with telomere maintenance mechanisms (*TERT* rearrangements, *ATRX* deletions, alternative lengthening of telomeres) have particularly dire prognoses with probabilities of <20% event-free and overall survival [11]. Novel frontline therapy elements beyond those successfully implemented and optimized by SIOPEN and COG are urgently warranted. High-risk neuroblastomas driven by *MYCN* amplifications and *ALK* alterations [12, 13] are similarly difficult to treat in healthcare systems where ALK inhibitor therapy is not yet available.

Mounting evidence shows consolidation therapy with single high-dose chemotherapy followed by autologous stem cell rescue does not benefit patients with high-risk neuroblastoma refractory to induction chemotherapy and salvage chemo-immunotherapy. Patients also suffer therapy-related acute and long-term side effects. Objective parameters for stringent evaluation at the end of induction chemotherapy and salvage chemo-immunotherapy have been defined. Most likely, tumor biology is heterogenous in these patients, necessitating a move to a novel therapeutic strategy assessed within early clinical trials.

A small patient subset fails to mobilize enough autologous hematopoietic stem cells for rescue after high-dose consolidation chemotherapy [14]. The high-dose chemotherapy regimen that significantly improves event-free and overall survival in patients with high-risk neuroblastoma cannot be administered to this patient subset. Advanced consolidation strategies for high-risk neuroblastoma with proven feasibility, safety and efficacy in the relapse setting could benefit patients (i) with tumors driven by telomere maintenance mechanisms combined with mutations in RAS/TP53 signaling; (ii) with tumors refractory to current state-of-the-art induction therapies and (iii) whose hematopoietic stem cells cannot be mobilized.

Haploidentical stem cell transplantation (haplo-SCT) with ex vivo TCRαβ/CD19-depleted grafts is a feasible, safe and effective option for patients with relapsed high-risk neuroblastoma [15, 16]. This approach enables rapid immune reconstitution, potential graft-versus-tumor benefit and is associated with a low incidence of graft-versus-host disease (GvHD) [15, 16]. Most importantly, early reconstitution of donor-derived natural killer cells (maximum day +14 to +30) after haplo-SCT elicits high antibody-dependent cytotoxicity in the context of GD2-directed immunotherapy [17]. The pilot cohort we report summarizes our experience with haplo-SCT and GD2-directed immunotherapy in the frontline setting for patients with high-risk neuroblastoma that fulfill at least one of the molecular or clinical parameters outlined above. Because busulfan/melphalan is more efficacious than platinum-based conditioning regimens in the autologous setting [18], we adapted the conditioning regimen accordingly.

## Subjects and methods

### Pilot cohort subjects

Five pediatric patients were diagnosed with INRG stage M high-risk neuroblastoma at *Charité— Universitätsmedizin Berlin* or *Helios Klinikum Berlin Buch* between 2017 and 2024. No phase III clinical trials treating high-risk neuroblastoma were open for patient recruitment in Germany between January 2017 and July 2023. The patient diagnosed most recently (2024) did not fulfill the inclusion criteria for the HR-NBL2 SIOPEN phase III clinical trial (NCT04221035) that opened at *Charité—Universitätsmedizin Berlin* in August 2023. All patients were enrolled in the neuroblastoma haplo-SCT registry (ethics approval 155/2018/BO2). Written informed consent was obtained from all parents or legal guardians, and data collection was conducted in accordance with institutional ethical standards.

### Clinical endpoints and statistical analysis

As primary objectives, we evaluated the (i) feasibility, defined as ≥95% engraftment, and (ii) safety, operationalized as <10% 100-day non-relapse mortality, of primary haploidentical stem cell transplantation (haplo-SCT) combined with dinutuximab beta as first-line consolidation therapy for genetically and/or clinically defined high-risk neuroblastoma at particularly high risk for unfavorable outcome as defined in Section 3.1. Non-relapse mortality encompassed death from any cause other than a disease relapse. Immune reconstitution, toxicity, acute and chronic graft-versus-host disease, as well as event-free and overall survival, were assessed as secondary objectives. The first of three consecutive days with an absolute neutrophil count >0.5 × 10⁹/L defined the day of engraftment. Primary graft failure was characterized by failure to achieve an absolute neutrophil count of 0.5 × 10⁹/L by day +28 after transplantation. Acute and chronic GvHD were graded according to established consensus criteria [19, 20]. Overall survival was calculated as the time from haplo-SCT until death by any cause. Progression-free survival was defined as survival without detection of disease progression or relapse at any time after transplantation. Event-free survival (EFS) was calculated from the time of initial neuroblastoma diagnosis until relapse, progression or last follow-up, whichever occurred first.

### Infection prophylaxis

Infection prophylaxis followed the institutional protocol and included antifungal agents (e.g., liposomal amphotericin B, azoles), cotrimoxazole to prevent *Pneumocystis jirovecii* pneumonia and antiviral agents (ganciclovir, foscavir, valganciclovir or acyclovir) administered according to donor-recipient viral serostatus (e.g., cytomegalovirus).

## Results

### Pilot cohort characteristics

Five patients (60% males, 40% females) ranging from 0 to 49 months at diagnosis (median age: 34 months, 2.8 years; 40% *MYCN*-amplified cases; Table 1) fulfilled at least one of three criteria for pilot cohort eligibility: (i) Tumor tissue obtained at initial biopsy demonstrated (targeted sequencing pipeline [21] applied) at least one *RAS-MAPK* pathway and/or *TP53* mutation combined with telomerase activation or alternative lengthening of telomeres [11] (Table 1). (ii) Disease failed to respond to induction treatment and was declared refractory (Table 2) as defined in the SIOPEN HR-NBL2 clinical trial (NCT04221035) protocol by a planar [^123^I]MIBG image with SIOPEN skeletal score >3 and/or less than a partial response according to International Neuroblastoma Response Criteria (INRC) [22]. (iii) A very young patient age (and correspondingly low body weight preventing peripheral CD34+ stem cell apheresis) or a failure to mobilize autologous stem cells during at least two G-CSF and plerixafor mobilization attempts [23]. Mobilization failure was defined by peripheral blood hematopoietic cell counts with a cumulative yield <1 × 10⁶ CD34+ cells/kg body weight.

**Table 1:** Baseline clinical and molecular characteristics of the pilot cohort comprised of five patients with high-risk neuroblastoma.

P#	Age at diagnosis (Months)	Sex	Stage (INRG)	Primary tumor site	Bone marrow metastasis	Metastatic soft tissue and bone disease	*MYCN* Status^a^	Other genetic markers^b^
1	33	M	M	SC, left	yes	yes	1	*TERT*-rearr. *TP53* c.638 G > A *ALK* c.3691 C > T^c^ FGFR4 c.1586 g > A^c^
2	34	M	M	SC, right	yes	yes	1	*ATRX* inactivating alteration; *CCND1* amplification; *ATM* deletion; *TSC1* c.3475 A > C^c^; *RICTOR* c.2120 A > G^c^
3	45	F	M	SC, left	yes	yes	2	1p36 imbalance *ALK* c.3522 C > A^c^
4	49	M	M	AG, right	yes	yes	1	*TERT*-rearr. *MTOR* c.1870C>T^c^ *ERBB2* c.877 G > A^c^
5	0	F	M	AG, bilateral	yes	yes	2	1p36 unknown ROS1 c.452 C > T^c^ TSC2 c.5116CC>T^c^ CREBBP c.1369 A > G^c^

*AG* adrenal gland, *ALK* anaplastic lymphoma kinase, *BM* bone marrow, *INRG* International Neuroblastoma Risk Group, *mut* mutant, *P#* patient number, *rearr* rearranged, *SC* sympathetic chain, *TERT* telomerase reverse transcriptase, *TP53* tumor protein p53.

^a^1, diploid; 2, amplified; analyzed by fluorescence in situ hybridization and next-generation sequencing.

^b^55 genes analyzed by targeted hybrid capture sequencing [21].

^c^variants with unknown functional significance.

**Table 2:** Overview of induction therapy, serial response assessment according to International Neuroblastoma Response Criteria and rationale for first-line haploidentical stem cell transplantation in our pilot cohort.

P#	[^123^I]-MIBG SIOPEN skeletal/soft tissue scores^a^ at initial diagnosis	Induction polychemo- therapy^a^	ALK inhibitor during induction therapy	[^123^I]-MIBG SIOPEN skeletal/soft scores^a^ at end of induction	Salvage chemo immuno- therapy^a^	[^123^I]-MIBG SIOPEN skeletal/soft scores^a^ after salvage therapy	Overall response prior to haplo-SCT^b^	Rationale for haplo-SCT in first-line therapy	[^131^I]-MIBG therapy activity (MBq/kg)
1	46/3	3xN5, 3xN6	Ceritinib	0/0	/	/	CR	Genetic tumor profile	/
2	2/1	3xN5, 3xN6	/	0/0	4x IT-DB	0/0	PR	Genetic tumor profile	444
3	5/1	3xN5, 3xN6	/	0/1	/	/	MR^e^	Genetic tumor profile	/
4	55/1	3xN5, 3xN6	/	41/1	4x IT-DB	10/1	MR	Refractory disease^c^	444
5	6/2	4xN4, 1xN5, 1xN6	/	4/2	4x IT-DB	0/0	MR^e^	Non-mobilizer^d^	/

*CR* complete response, *DB* dinutuximab beta, *haplo-SCT* haploidentical stem cell transplantation, *IT* irinotecan-temozolomide chemotherapy backbone, *MIBG* metaiodobenzylguanidine, *MR* minor response, *PR* partial response.

^a^Patients were treated according to German national therapy recommendations [24] with respect to induction (salvage) therapy and primary tumor surgery. N4, N5 and N6 refer to polychemotherapy cycles outlined in the German national therapy recommendations. The SIOPEN scoring system is outlined for both skeletal and soft tissue lesions and is based on planar [^123^I]MIBG imaging, in line with recommendations from the SIOPEN HR-NBL2 clinical trial protocol (NCT04221035). The SIOPEN soft tissue score aggregates assessments across eight soft tissue segments covering the trunk and extremities, each graded from 0 to 2 according to the number of MIBG-avid metastatic lesions within the segment (excluding the primary tumor), yielding a maximum total score of 16. The SIOPEN skeletal score quantifies the extent of MIBG-avid skeletal lesions by assigning a score of 0 to 6 to each of 12 skeletal segments, resulting in a maximum total score of 72.

^b^According to the International Neuroblastoma Response Criteria [22].

^c^As defined in the SIOPEN HR-NBL2 clinical trial protocol (NCT04221035).

^d^Non-mobilizer refers to a case in which autologous stem cells could not be mobilized due to the low body weight in patient with a very young age.

^e^In patients 3 and 5, the overall response prior to haploidentical SCT was classified as a minor response according to the International Neuroblastoma Response Criteria [22], due to persistent minimal disease in the bone marrow. The response of the primary (soft tissue) tumor and of metastatic soft tissue and bone lesions were partial response (PR) in patient 3 and complete response (CR) in patient 5, respectively. In patient 2, residual primary soft tissue tumor resulted in an overall partial response before haplo-SCT.

### Overall response prior to haplo-SCT

Induction polychemotherapy was administered according to national therapy recommendations [24]. N5 (vindesine, cisplatin, etoposide) and N6 (vincristine, dacarbazine, ifosfamide, doxorubicin) cycles were alternated for a total of six cycles (Table 2). Patient 5 received four N4 cycles (cyclophosphamide, vincristine, doxorubicin), due to young patient age, followed by N5 and N6 cycles (Table 2). The primary tumor was resected after three to five induction therapy cycles at a national reference center for high-risk neuroblastoma surgery. Inoperable residual primary tumor tissue in the *coeliac trunc* region was documented by MRI and [^123^I]MIBG scintigraphy in Patient 2 and by MRI in Patient 4 (Table 3). Achieving full gross total tumor resection required two operations in Patient 3 (Table 3). Salvage chemo-immunotherapy consisted of GD2-directed immunotherapy (dinutuximab beta) combined with an irinotecan-temozolomide chemotherapy backbone (IT-DB), and was administered to patients with insufficient tumor response at metastatic soft tissue and bone sites or partial primary tumor response (determined by INRC [22] at the end of induction). The Children’s Oncology Group data published by Mody et al. [25] was used at the time to design the salvage chemo-immunotherapy regimen applied. Results from the ITCC-SIOPEN BEACON Immuno Phase II trial (NCT02308527) [26] and the French national SACHA registry (NCT04477681) [27] have since confirmed this regimen to be well-tolerated and effective in approximately 40% of patients with relapsed or refractory high-risk neuroblastoma. Patients received four cycles of salvage chemo-immunotherapy based on responses assessed after two and four IT-DB cycles (Table 2). Prior to haplo-SCT, one patient each (20%) achieved complete remission or a partial response, and three patients (60%) achieved minor responses (Table 2). Planar [^123^I]MIBG imaging in Patient 4 had a SIOPEN skeletal score >3. Overall responses assessed prior to haplo-SCT varied among patients even in this small pilot cohort, suggesting response can be broad for patients to whom this therapeutic course is selected.

**Table 3:** Local therapy concepts for patients with high-risk neuroblastoma in the pilot cohort.

P#	Primary tumor resection	Primary tumor response^a^	Primary tumor site proton therapy^a^	MIBG-avid metastasis proton therapy^a^
1	Gross total tumor resection	CR	no^b^	no
2	Gross total tumor resection	PR	21.6 Gy + boost 14.4 Gy	no
3	Gross total tumor resection and second-look surgery	PR	21 6 Gy + boost 14.4 Gy	no
4	Adrenalectomy	PR	21.6 Gy + boost 14.4. Gy	no
5	Adrenalectomy with atypical partial hepatectomy	CR	/	/

*CR* complete response, *MIBG* metaiodobenzylguanidine, *PR* partial response.

^a^Patients were treated according to German national therapy recommendations [24]. Primary tumor response was assessed by multimodal imaging at day +30 after haplo-SCT.

^b^At the time of treatment, German national therapy recommendations [24] did not recommend local radiotherapy of the primary tumor region.

### Successful primary engraftment after busulfan-based haplo-SCT

Patients with [^123^I]MIBG-positive lesions immediately following salvage chemo-immunotherapy, and qualifying to proceed to haplo-SCT, received [^131^I]MIBG therapy based on the reported benefit of combining [^131^I]MIBG therapy with haplo-SCT in the relapse setting [15]. Patients 2 and 4 tolerated the 444 MBq/kg ( = 12 mCi/kg) [^131^I]MIBG [28–30] received well. Patients without mIBG-avid disease in planar [^123^I]MIBG imaging were ineligible for [^131^I]MIBG therapy. All five patients underwent a myeloablative conditioning regimen comprising busulfan (Bu, target area under the curve, AUC, 70–95 mg × h/L), melphalan (Mel, 90–140 mg/m²), fludarabine (Flu, total 160 mg/m²) and antithymocyte globulin (ATG, total 45–60 mg/kg infused prior to Bu/Mel/Flu; Table 4). Overall, patients tolerated ATG well. Busulfan dosing was adapted from the SIOPEN HR-NBL1 trial (NCT01704716) protocol as needed to maintain the target exposure, and AUC for individual busulfan plasma concentration was calculated by non-compartmental analysis applying a log-linear trapezoidal method [31, 32]. The median busulfan AUC was 88 mg × h/L (range: 77–90 mg × h/L; Table 4). Melphalan was administered intravenously following the completion of the busulfan infusion. After conditioning, all patients received infusions of haplotype-matched, ex vivo T-cell receptor (TCR) αβ/CD19-depleted peripheral blood stem cells. All peripheral blood stem cell donors were mismatched parents (4 fathers, 1 mother) with ≥4 allele disparities (4–5 loci mismatches). Stem cell products were depleted of TCRαβ^+^ and CD3^+^ T cells and CD19^+^ B cells using an automated CliniMACS^®^ Plus device (Miltenyi Biotec, Bergisch Gladbach, Germany). Grafts were engineered to contain ≥4 × 10^6^ CD34^+^ progenitor cells/kg recipient body weight, while limiting TCR αβ^+^ T cells to ≤25 × 10^3^ and CD19^+^/CD20^+^ B cells to ≤1 × 10^5^ cells/kg recipient body weight (Table 5). [33] GvHD prophylaxis consisted of a short mycophenolate mofetil course (2 × 600 mg/m² daily from day –1 to +30 post-transplantation; Table 4), enabling timely GD2-immunotherapy while minimizing the risk of early acute GvHD. Primary engraftment was achieved in all patients, with a median time to neutrophil engraftment of 10 days (range, 8–14 days). Thus, no primary graft failure occurred, meeting the primary endpoint ( ≥ 95% engraftment) for the entire pilot cohort. Chimerism data documented robust and stable engraftment quality as previously published (Supplementary Table S1). [33]

**Table 4:** Conditioning regimen, drug dosage and therapeutic drug monitoring data for pilot cohort.

P#	Conditioning Regimen^a^	Bu AUC (ng x h/ml)	Mel (mg/m^2^)	Flu (mg/m^2^)	ATG (mg/kg)	Graft	Donor	Immuno-suppression; stop (day)
1	Bu/Mel/Flu/ATG	90,000	120	160	60	TCRα/β CD19-depleted PBSCs	M	MMF; d + 30
2	Bu/Mel/Flu/ATG	88,000	120	160	60		F	
3	Bu/Mel/Flu/ATG	77,000	120	160	60		F	
4	Bu/Mel/Flu/ATG	89,000	120	160	60		F	
5	Bu/Mel/Flu/ATG	77,260	100	100	45		F	

*ATG* antithymocyte globulin, *Bu* busulfan, *F* father, *Flu* fludarabine, *M* mother, *Mel* melphalan, *MMF* mycophenolate mofetil, *P#* patient number, *PBSCs* peripheral blood stem cells, *TCR* T-cell receptor.

^a^The conditioning regimen used combined myeloablative Bu/Mel [18] with fludarabine and ATG. Busulfan dosing was guided by therapeutic drug monitoring targeting an area under the curve (AUC) between 70,000 and 95,000 ng x h/ml [31]. Starting dosages were in line with the recommendation by Nguyen et al. [48] and the SIOPEN HR-NBL1 trial protocol. Melphalan was administered at a total dose of 120 mg/m^2^, reflecting the use of a combination of two myeloablative agents (busulfan and melphalan) to limit cumulative regimen-related toxicity. Busulfan clearance and exposure exhibit substantial inter-individual and intra-individual variability, making initial dosing and prediction of therapeutic levels challenging. Please note that this unpredictability is particularly pronounced in neonates and infants, where maturation of metabolic pathways, organ function and body weight significantly affect busulfan pharmacokinetics. Nonetheless, determining exposure via therapeutic drug monitoring remains preferable to no monitoring. [49–51] Fludarabine dosing was chosen according to Bethge et al. [33], and antithymocyte globulin (ATG Grafalon) was given as graft rejection prophylaxis in line with the institutional standard of care. Dosages for patient 5 were reduced due to young age and low body weight.

**Table 5:** Graft composition overview for pilot cohort.

P#	CD34^+^CD45^+^ cells (x10^6^/kg)	CD3^+^ cells (x10^6^/kg)	TcR αβ^+^ cells (x10^5^/kg)	TcRγδ^+^ cells (x10^9^/kg)	CD19^+^/CD20^+^ cells (x10^5^/kg)	NK cells (x10^9^/kg)
1	28.48	25.12	0.1150	0.025	1.017	0.058
2	35.19	18.48	0.2622	0.018	0.340	0.101
3	50.54	5.56	0.1499	0.005	0.677	0.272
4	39.49	64.6	0.0433	0.065	0.554	0.148
5	9.65	54.03	0.2949	0.054	1.381	0.082

*CD34*^+^* CD45*^+^ hematopoietic stem cells expressing the CD34 and CD45 antigens, *TcRαβ*^+^ T-cell receptor alpha-beta-positive T cells, *CD3*^+^ all T lymphocytes, *CD19*^+^*/CD20*^+^* B lymphocytes* TcRγδ^+^ T-cell receptor gamma-delta-positive T cells, *NK cells* natural killer cells, *P#* patient number.

### Complications after transplantation

Patient 5 experienced partial respiratory insufficiency 18 days after haplo-SCT. *Pneumocystis jirovecii* pneumonia was diagnosed by the infectious work-up, and rapidly resulted in acute respiratory distress syndrome requiring venovenous extracorporeal membrane oxygenation. Despite the immediate start of antibiotic therapy and continuous maximal intensive care measures, pulmonary function could not be restored. Postulating an additional drug-induced lung injury (despite closely monitored nontoxic busulfan levels; Table 4), we administered a corticosteroid pulse that did not improve parameters. Patient 5 died 36 days after haplo-SCT (day +8 of venovenous extracorporeal membrane oxygenation).

Early post-transplant viral reactivation occurred for Epstein-Barr virus in one patient (20%), BK virus (also known as human polyoma virus) in another patient (20%), adenovirus in stool samples from three patients (60%, viremia in one patient) and HHV-6 in one patient (20%). Cytomegalovirus reactivation was not detected in any patient. Acute grade I GvHD of the skin (not requiring systemic therapy) occurred in one patient (20%). Chronic GvHD and lymphoproliferative disorders were not observed. Grade ≥3 mucositis was the most frequent therapy-related adverse event during haplo-SCT, affecting 4 patients (80%). Moderate veno-occlusive disease (that fully resolved) occurred in one patient (20%). Non-relapse toxic mortality before day +100 occurred in 1 (20%) haplo-SCT patient, so that the second primary endpoint ( < 10% non-relapse mortality) was not met in the pilot cohort.

### Immune recovery after haplo-SCT and its usage for GD2-directed consolidation therapy

**Fig. 1:**
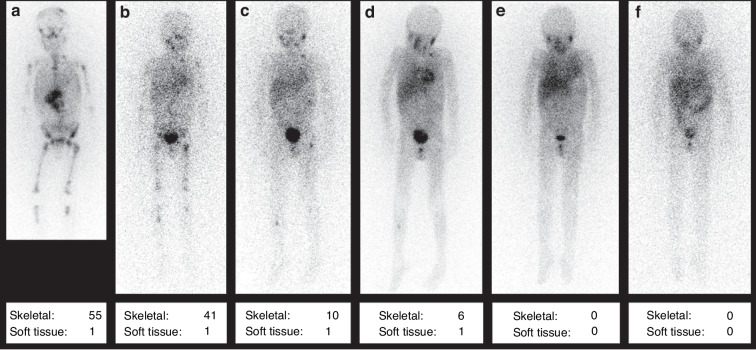
Serial planar [^123^I]MIBG imaging of Patient 4 with refractory high-risk neuroblastoma. Shown are the skeletal and soft tissue SIOPEN scores for timepoints A-F derived from the planar 24-hour images. Timepoint **a** initial diagnosis; **b** end of induction polychemotherapy; **c** end of salvage chemo-immunotherapy; **d** after [^131^I]MIBG therapy and haplo-SCT; **e** after 3 dinutuximab beta immunotherapy cycles; **f** after 6 dinutuximab beta immunotherapy cycles.

Immune recovery following T cell-depleted haplo-SCT showed the characteristic predominance of natural killer cells (CD56^+^) in the early phase (Table 6; day +30). Progressive T cell (CD3^+^, αβ^+^ and γδ^+^) and B cell (CD19^+^) recovery was evident by day +100 (Table 6), reflecting adaptive immune compartment restoration. Starting earliest on day +31 after discontinuing immunosuppressive mycophenolate mofetil therapy, patients received a total of six 35-day dinutuximab beta cycles (10 mg/m^2^/day for 10 days followed by a 25-day break). Treatment-associated pain and cytokine release syndrome during cycle 1 and decreasing dinutuximab-associated pain as the cycle number increased were universal. No additional interleukin 2 was administered. Patient 3 with *ALK*-altered neuroblastoma received ALK inhibitor therapy (3 months lorlatinib [34], discontinued due to neurological symptoms; 21 months ceritinib [35]) in parallel to GD2-directed immunotherapy. Local proton radiotherapy of the primary tumor site followed national recommendations [24], and was administered from day +60 onwards after haplo-SCT in patients with a metastatic complete response (Patients 2 and 3) and postponed to consolidation therapy end in Patient 4 (metastatic minor response; Fig. 1). Patient 4 started [^131^I]MIBG therapy with active disease (comprising 11 MIBG-avid metastases in planar imaging), but entered follow-up in complete metastatic remission documented by [^123^I]MIBG diagnostics (Fig. 1). After completing consolidation therapy, Patient 3 received as maintenance a GD2-directed vaccination therapy [36].

**Table 6:** Cellular immune reconstitution in pilot cohort at days + 30 and + 100 after haploidentical stem cell transplantation.

Cell counts^a^	CD3^+^/ µl	CD4^+^/ µl	CD8^+^/ µl	CD19^+^/ µl	CD56^+^/ µl	α/β T cells/µl	γδ^+^ cells T cells /µl	Lympho- cytes/µl	Leuko- cytes/µl
**Day** + **30**									
median	55	10	10	40	280	10	45	475	3425
min	30	10	0	10	200	10	20	260	2900
max	80	10	30	140	620	30	70	750	7390
**Day** + **100**									
median	565	140	105	300	235	395	125	1050	5435
min	110	30	20	200	80	20	50	590	3020
max	770	240	360	1300	460	590	290	2450	7890

^a^Absolute counts for CD3^+^, CD4^+^, CD8^+^, CD19^+^, and CD56^+^ lymphocyte subsets, αβ^+^ and γδ^+^ T cells, total lymphocytes and leukocytes are reported as median (med), minimum (min) and maximum (max) values.

### Patient survival in the pilot cohort

**Fig. 2:**
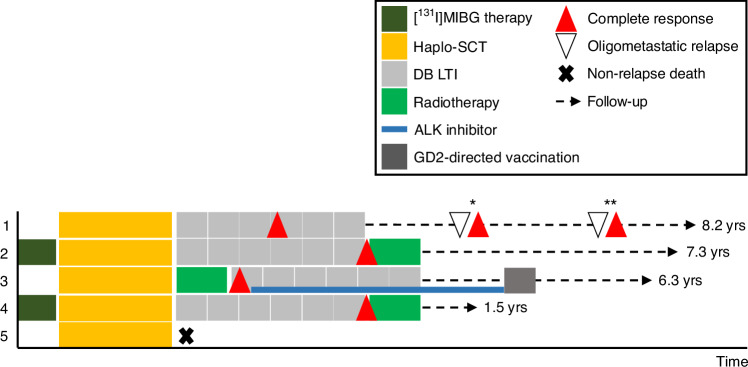
Swimmer plot of treatment response in the pilot cohort. Shown are the individual follow-up periods for each patient (patient numbers listed to the left). DB, dinutuximab beta; Haplo-SCT, haploidentical stem cell transplantation; LTI, long-term infusion; MIBG, metaiodobenzylguanidine; yrs, years. *First relapse therapy consisted of reinduction therapy, 2nd haplo-SCT with GD2 antibody and GD2-directed vaccination therapy. **Second oligometastatic relapse therapy consisted of local therapy (surgery, radiotherapy) followed by dinutuximab beta long-term infusion.

Patient 1 experienced first oligo-metastatic relapse 3.11 years after initial diagnosis. Chemo-immunotherapy and a second haplo-SCT followed by GD2-directed immunotherapy combined with checkpoint inhibition achieved a second complete remission (Fig. 2). The second oligometastatic relapse (4.3 years after first relapse) was treated with local (surgery, radiotherapy) and systemic (dinutuximab beta monotherapy) measures. Patient 1 maintains a third complete remission at data cut-off (December 31, 2025) 8.2 years after initial high-risk neuroblastoma diagnosis (*TERT*-rearranged and *TP53*- and *ALK*-mutated, Fig. 2). Patients 2-4 have remained in first complete remission at 7.3, 6.3 and 1.5 years follow-up, respectively (Fig. 2). Patient 5 experienced non-relapse death 36 days after haplo-SCT (Fig. 2). Altogether, the pilot cohort demonstrated 80% overall, 80% progression-free and 60% event-free survival.

## Discussion

We aimed with this pilot study to evaluate the feasibility and safety of combining primary busulfan-based haploidentical stem cell transplantation (haplo-SCT) with anti-GD2 immunotherapy in patients at particularly elevated risk for unfavorable neuroblastoma outcome. The haplo-SCT regimen combined with post-consolidation GD2-directed immunotherapy was designed to harness a potential graft-versus-tumor effect, provide the patient with a new healthy immune system and provide direct antitumor effects through the intense conditioning chemotherapy previously shown to be more effective than other high-dose myeloablative drug combinations [18]. Our findings highlight an overall favorable, feasible profile, affirming that this intensive therapeutic approach can be effectively delivered in a very high-risk pediatric population. Our inclusion criteria were designed to select clinically challenging populations for whom conventional therapies currently offer only limited durable remission and in whom we could test haplo-SCT applicability. These were patients refractory to induction therapy or failing to mobilize peripheral hematopoietic stem cells, and patients with very high-risk tumors harboring *RAS*/*TP53* pathway mutations combined with a mechanism to maintain telomeres. Although the EFS in this pilot cohort appears favorable compared to the ~20% EFS in matched historical cohorts, these results must be interpreted with caution, given the small pilot sample size and non-randomized design. Efficacy endpoints (e.g., overall, progression-free or event-free survival) were not the primary objectives in this pilot cohort, which limits conclusions about long-term clinical benefits.

The busulfan/melphalan-based conditioning regimen, adapted from the SIOPEN HR-NBL1 trial (NCT01704716) and combined with fludarabine and ATG, was well-tolerated. Rapid engraftment and low rates of severe toxicity and GvHD were observed, likely reflecting the large number of transfused stem cells, profound T-cell depletion and graft engineering strategies employed [33, 37]. We observed one case of lethal respiratory failure, clinically manifesting on day +18 in a 12-month-old child in our cohort. Despite current standard-of-care chemo-immunotherapy dosage prior to haplo-SCT and maintenance of adequate busulfan serum levels, pulmonary function did not recover, as is usually observed for this well-documented breakthrough infection in the transplant setting, after successfully treating *Pneumocystis jirovecii* pneumonia. The lack of recovery may be linked to toxic effects induced by the combination of infectious and toxic mediators of lung injury, and was possibly aggravated by the young patient's age and low body weight. While the potential multifactorial causes and sequelae leading to this isolated irreversible respiratory failure could not be resolved, they resemble other recent case reports in the context of multimodal neuroblastoma therapy [38]. Altogether, we regard pneumonia caused by *Pneumocystis jirovecii* pneumonia in the early post-transplant phase as the most likely cause of the acute respiratory distress syndrome. Nevertheless, we decided that small infants will be excluded from future iterations until more experience is gained.

Immune reconstitution in this pilot cohort was consistent with previous experience with pediatric haplo-SCT with T cell-depleted grafts [15, 16], demonstrating the early recovery of natural killer cells followed by progressive restoration of adaptive immunity. None of the viral reactivations/primary infections resulted in clinically significant disease, reflecting effective infection control through rigorous screening, antiviral prophylaxis, prompt therapeutic intervention and overall management within an experienced stem cell transplantation center. The robust natural killer cell recovery likely contributed to this favorable safety profile and supports the mechanistic rationale for combining haplo-SCT with GD2-directed immunotherapy to enhance antibody-dependent cytotoxicity. The omission of interleukin 2 from the consolidation regimen was consistent with recent evidence demonstrating increased toxicity without added benefit [39], and contributed to the manageable toxicity profile observed.

Several interesting alternative approaches to treat children with refractory or relapsed high-risk neuroblastoma are currently being investigated. The MINIVAN phase I NCT02914405 clinical trial investigated combining [^131^I]-MIBG radiotherapy with the PD-1 checkpoint inhibitor, nivolumab, and dinutuximab beta. Preclinical data suggested this multimodal strategy could achieve synergistic anti-tumor and immunomodulatory effects [40]. Initial trial data suggest an overall response rate (partial and complete responses) of 42.9% (12/28 patients) [41]. Promising results have been achieved with GD2-targeting CAR T cells in patients with relapsed or refractory disease [42]. The pivotal phase I-II trial (NCT03373097) demonstrated the feasibility, safety and manageable toxicity profiles of autologous GD2-CART01, which achieved durable responses with persisting CAR T cell detection, particularly in patients with low disease burden. Although patients with neuroblastomas having a very high-risk genetic profile [11] were not explicitly reported in this trial cohort, such molecularly defined subgroups within the overall high-risk population may benefit from this [42] or other cellular therapy approaches [43, 44]. For poor responders to frontline induction therapy, tandem autologous stem cell transplantation, as reported by COG [45] and currently evaluated in the SIOPEN HR-NBL2 clinical trial (NCT04221035), may improve EFS compared to single transplantation, underscoring the value of intensified consolidation for patients with insufficient initial response. Patients failing to achieve a sufficient metastatic response at the end of salvage chemo-immunotherapy are in a particularly challenging situation, given the potential for high cumulative treatment toxicity and persistent inadequate disease control. Patient 4 in our pilot cohort is a representative of this very vulnerable patient subset, whose active refractory disease responded to our therapy concept.

Haplo-SCT employing ATG/Flu/TT/MEL is the standard of care for patients with relapsed high-risk disease in Germany [15]. Graduating this successful strategy from the relapse to the frontline setting, as evaluated for a growing list of other options [46], could provide an effective foundation for frontline therapy through its enhanced antibody-dependent cytotoxicity and graft-versus-tumor effects. This approach may also facilitate subsequent donor-derived CAR T cell therapy use in the event of relapse [47], thus providing a flexible and potent immunotherapeutic platform for patients with high-risk neuroblastoma. Nonetheless, caution should be used when interpreting outcomes due to the small size and patient heterogeneity in our pilot cohort. A prospective study with a larger sample size and adequate power is needed to comprehensively evaluate the balance between safety, tolerability and long-term clinical efficacy of this approach. In conclusion, our data suggest that primary busulfan-based haplo-SCT combined with GD2-directed immunotherapy is a feasible and effective consolidation strategy to treat subsets of patients with high-risk neuroblastoma.

## Supplementary information

Supplemental Material

## Data Availability

All data generated or analyzed during this study are included in this published article [and its supplementary information files].
