# Supplementary material for: A pilot study combining primary busulfan-based haploidentical stem cell transplantation with GD2 antibody to treat high-risk neuroblastoma
Source: Bone Marrow Transplant. 2026 May 29;61(7):926–34. doi: 10.1038/s41409-026-02912-2 (PMC13349867; doi:10.1038/s41409-026-02912-2)
Supplement: Supplementary file 1 — Supplemental Material [file 41409_2026_2912_MOESM1_ESM.docx]

**SUPPLEMENTARY DATA FOR**

**A pilot study combining primary busulfan-based haploidentical stem cell transplantation with GD2 antibody to treat high-risk neuroblastoma**

Sveva Castelli^*^, Tim Flaadt^*^, Franziska Schulze, Theresa M. Thole-Kliesch, Felix Zirngibl, Lena Oevermann, Annette Künkele, K. Astrahantseff, Stefanie Schulte, Louisa Duell, Joerg Fuchs, Thorsten Simon, Barbara Hero, Patrick Hundsdoerfer, Julian M.M. Rogasch, Peter Lang, Steven Warmann, Arend von Stackelberg, Johannes H. Schulte^†^, Angelika Eggert^†^, Hedwig E. Deubzer^†^

*** *These first authors contributed equally to this work. † These senior authors contributed equally to this work.*

**Running title:** Frontline haplo-SCT with GD2 antibody

**Table of Contents**

Supplementary Table S1 starting p. 2

**Supplementary Table S1. Next-generation sequencing-based chimerism monitoring.**

| Days after transplantation | Sample | Total chimerism (%)^1^ | CD34^+^ (%)^2^ | CD3^+^ (%)^3^ |
| --- | --- | --- | --- | --- |
| ***Patient 1*** | | | | |
| 27 | Blood | 100 | n.a. | n.a. |
| 57 | Blood | 100 | n.a. |  |
| 100 | Bone marrow | 100 | 94 | 100 |
| 181 | Blood | 100 | 100 | 100 |
| 264 | Bone marrow | 100 | 100 | 100 |
| ***Patient 2*** | | | | |
| 29 | Blood | 100 | n.a. | n.a. |
| 51 | Blood | 100 | n.a. | n.a. |
| 150 | Blood | 100 | 100 | 100 |
| 259 | Blood | 100 | 100 | 100 |
| 339* | Bone marrow | 100 | 100 | 100 |
| ***Patient 3*** | | | | |
| 22 | Blood | 100 | 100 | n.a. |
| 70 | Blood | 100 | 100 | 100 |
| 99 | Bone marrow | 100 | 100 | 100 |
| 177 | Blood | 100 | 100 | 100 |
| 367 | Bone marrow | 100 | 100 | 100 |
| ***Patient 4*** | | | | |
| 31 | Blood | 100 | 100 | 100 |
| 64 | Blood | 100 | 100 | 100 |
| ***Patient 5*** | | | | |
| 19 | Blood | 98 | 85 | 98 |
| 26 | Blood | 99 | n.a. | n.a. |

^1^Shown is the proportion of donor cells in the total cell population.

^2^Shown is the proportion of donor cells in the CD34^+^ selected cell population.

^3^Shown is the proportion of donor cells in the CD3^+^ selected cell population.

n.a., not analyzed.
